# An Internet-Based Simulation Model for Nitrogen Management in Agricultural Settings

**DOI:** 10.1100/tsw.2001.337

**Published:** 2001-11-14

**Authors:** M.J. Shaffer, B.J. Newton, C.M. Gross

**Affiliations:** USADA Agricultural Research Sevice, Fort Collins, CO 80522, USA

**Keywords:** nitrate leaching, fertilizer, manure, soil nitrogen, nitrous oxide, greenhouse gas emissions, simulation model, ground water quality, NLEAP

## Abstract

Complex chemical, physical, and biological processes mediate nitrogen (N) transformations and movement during agricultural production, making the optimization of fertilizer use and environmental protection exceedingly difficult. Various computer models have been developed to simulate the site-specific fate and transport of N resulting from different crop production scenarios, but these models are very complex and difficult to use for most farmers, consultants, and conservationists. In an effort to facilitate access and simplify the use of sophisticated models, the U.S. Department of Agriculture (USDA) has developed an Internet-based nitrogen analysis tool. Based on the Nitrate Leaching and Economic Analysis Package (NLEAP), the Web site allows a user to conduct multiyear N simulation modeling specific to a crop field. Servers handle much of the required data assembly and formatting, thus sparing the user's resources. Model runs are executed on the servers and the results are transmitted to the user. This new tool is presented along with early implementation results.

## INTRODUCTION

Precise management of soil nitrogen (N) is critical for optimal crop production and environmental protection. N is the crop-growth limiting nutrient in most agricultural systems, and also presents considerable risk to the environment in terms of potential nitrate-N ($NO_3$-N) leaching to groundwater, N runoff and erosion losses to surface water bodies, and air pollution from volatilization of ammonia and nitrous oxide ($N_2O$) greenhouse gas emissions. The N cycle (Fig. 1) is quite complex, with many interrelated processes operating simultaneously[1]. The range of soil N sources includes commercial fertilizers, manure, other organics such as municipal and food processor wastes, crop residues (tops and roots), N fixed from the atmosphere by legumes and other organisms, and N introduced in precipitation and irrigation. Simulation models of the N cycle integrate the many soil N processes and inputs and provide management decision tools useful to farm managers, environmental consultants, action agencies, and scientists[2]. However, routine use of these tools by farmers, consultants, and conservationists has been restricted by difficulty of access and use, and limited database availability[3].

The Nitrate Leaching and Economic Analysis Package (NLEAP) model[4,5,6,7,8] was developed as a national effort to provide a simplified tool for estimating $NO_3$-N leaching from agricultural lands. The model has been utilized and tested extensively on a national and international basis with respect to leaching of $NO_3$-N from agriculture[9,10,11,12,13,14,15,16,17,18,19,20,21]. NLEAP is a mechanistic model that simulates soil-plant processes in one dimension, starting with crop residue and cropping effects on the soil surface and continuing down through the crop root zone to the bottom of the soil profile. The model was recently extended to include emissions of $N_2O$ greenhouse gases, organic residue decay on the soil surface, and variable rooting depths for crops[6,7,22]. NLEAP originally was delivered as a stand-alone package for use on PCs. NLEAP versions 1.2 and 1.3 contain a user interface with a DOS windowing system and regional databases for soils, climate, and crop parameters across the U.S. (http://www.gpsr.colostate.edu). These versions are limited to use on PC machines running DOS or Windows operating systems, or to DOS emulators on some other systems.

The recent emergence of the Internet is having an immense effect on the way modeling tools and information can be accessed and utilized on a global scale. Using the Internet, it should be possible for a user to access and effectively apply nutrient management and a range of other models from anywhere in the world and from a variety of computer hardware and software platforms. Client-server technology has also been developing rapidly and has become an integral part of Internet applications. Broad-based applications and databases (servers) can now be distributed at different locations and utilized when needed by the clients. Models and databases can also be maintained at locations where the key expertise resides, and the length of the code that must be downloaded into the client's computer memory over the Internet can be reduced. Clients can also have access to more expensive packages, such as geographic information systems (GIS), and have a greater choice of models and databases (servers) to use. In the extreme case in which the client (user) does not yet have access to the Internet, a version of the entire client-server modeling package can be provided as a stand-alone application on a single computer or internal network.

Linkage of Internet-based client-server technology with existing agricultural simulation models, databases, and GIS spatial programming offers the promise of improved ease of use, access, and maintenance for these tools.

The purpose of this paper is to describe the development of a client-server environment on the Internet for the analysis of $NO_3$-N leaching and $N_2O$ soil gas emissions. The goals of the NLEAP Internet server application are (1) easier access to the most recent NLEAP model, (2) easier and faster access to associated national databases for soils and climate, and (3) direct integrated access to GIS technology to facilitate the analysis of multiple soil and management combinations.

## METHODS

Internet-based client-server technology was used to develop support for the NLEAP simulation model and its associated databases. The Java programming language introduced by Sun Microsystems allowed for the development of applet interface windows that run in a Web browser such as Netscape and are somewhat independent of hardware and software platforms[23]. The Visual Café development environment (a graphical computer tool for Java programming[24]) was used to facilitate a more rapid development of individual Java applets. Connectivity between the Web browser interface and the servers was provided using File Transfer Protocol (FTP), Hypertext Markup Language (HTML), Practical Extraction and Report Language (PERL), Java, Common Gateway Interface (CGI), and Structured Query Language (SQL) methodology[23,25,26,27,28].

Extensive work with earlier DOS versions of NLEAP has demonstrated the need for long-term simulations (10+ years) that estimate $NO_3$-N leached (NL) at dynamic steady state[29]. This is needed to account for management differences in crop rotational sequences involving several different crops, and to remove the influence of initial soil conditions on simulated soil $NO_3$-N leached and $N_2O$ emissions.

Spatial variability in soils and topography across fields and farms coupled with recent interest in precision agriculture have demonstrated the necessity of handling multiple soils and associated management scenarios within field and farm settings and across local regions. The tools of most use here are GIS applications coupled with the simulation models that allow georectified

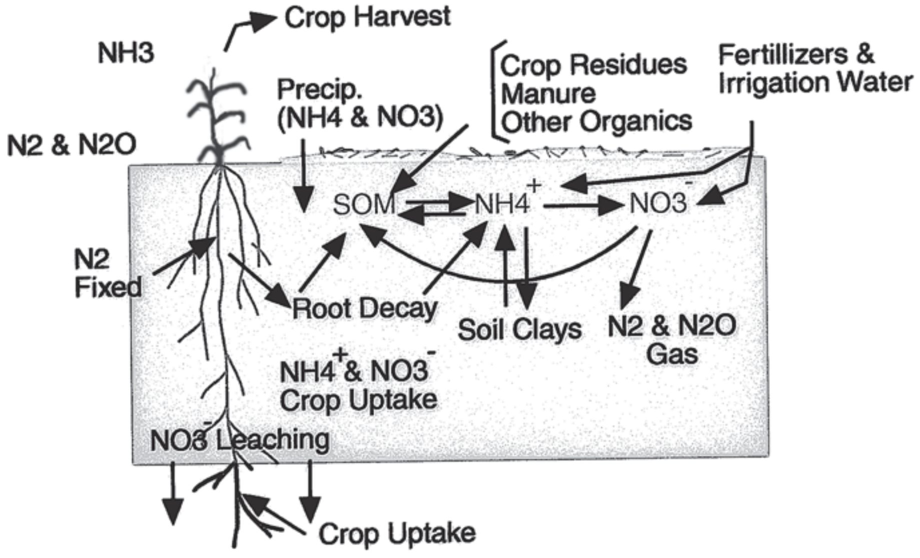

**FIGURE 1.** The nitrogen cycle.

layers of soil, climate, and management data to be mapped, simulated, and displayed through the use of specially designed GIS algorithms in conjunction with the models. Client-server GIS applications suitable for use on the Internet, such as ArcIms[30], are now available for use in conjunction with simulation models such as NLEAP.

Access to current soil and climate databases is important because these resources are dynamic and may change rapidly as new information becomes available. The USDA Natural Resources Conservation Service (NRCS) as well as other agencies (e.g., the National Oceanic and Atmospheric Administration [NOAA], the U.S. Environmental Protection Agency [EPA], and the National Aeronautics and Space Administration [NASA]) and private groups now have Web sites that make these types of resources available (e.g., http://www.wcc.nrcs.usda.gov).

## RESULTS AND DISCUSSION

The general configuration of the NLEAP client-server system for use on the Internet is shown in Fig. 2. The Java applet interface windows for use in the Netscape Web browser provide access to the NLEAP model server, the individual user account files on the NLEAP Web server, the NRCS soil and climate database servers, and the NLEAP ArcIms GIS server. The majority of the data flow occurs directly between the servers and the databases with the user interface acting as a control and display device. The user's account also contains configuration files allowing for customization of individual applet menu lists and the crop parameter file for multiple crops. The NLEAP client-server system was designed to minimize upload and download times and conserve usage of bandwidth over the Internet. Applet sizes have been kept small (75 KB to 1.5 MB) and data transfers between the client and servers have been limited to operational commands, minimal data uploads, and data downloads for summaries of graphical, GIS mapping, and tabular output. The vast majority of the computational work is performed by the servers.

A single Web page was created to provide access to the Java applet tools (Fig. 3). The applets are organized into quick analysis and detailed editing wizards located along the top of the computer screen, and basic and advanced tools and file utilities located along the left side. Each of these tools can be individually loaded by clicking with the mouse. The example in Fig. 3 shows the simulation and graph tools loaded along with a graphic output displaying $NO_3$-N leached over time.

Security restrictions for applets on the Internet became significant for cases where capability was provided for access to the user's computer for printing or file access. This was overcome by using signed applets and security certificates. This allowed the user to grant or deny access to the local (client) computer from the applet.

### Management Applets

Configuration of long-term management scenarios is accomplished using a two-tier approach in which preconfigured management may be selected using a set of menus containing typical combinations. Then a detailed management editor can be used to make site-specific changes and extensions to preprogrammed scenarios. Configuration of entire long-term scenarios is also possible using the detailed editor or an off-line editor such as the

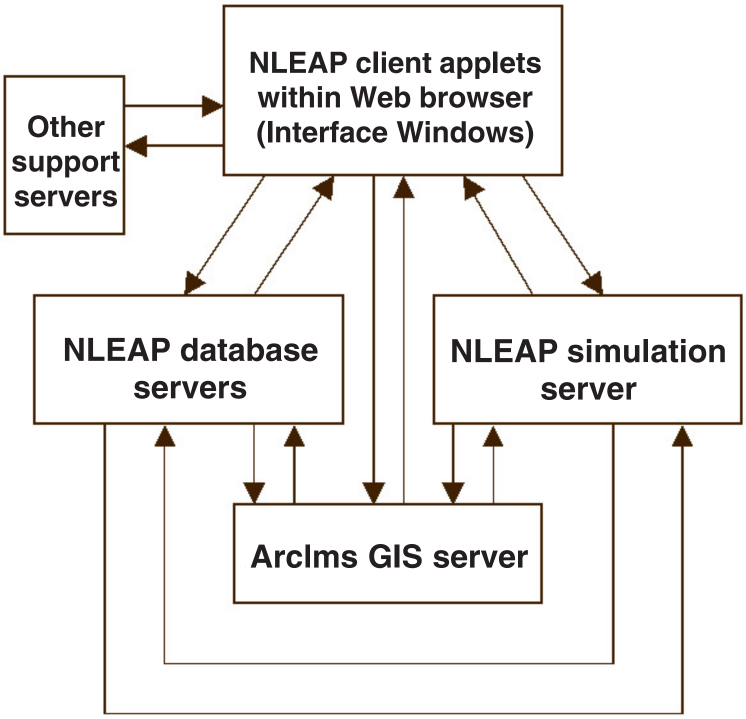

**FIGURE 2.** NLEAP client-server system.

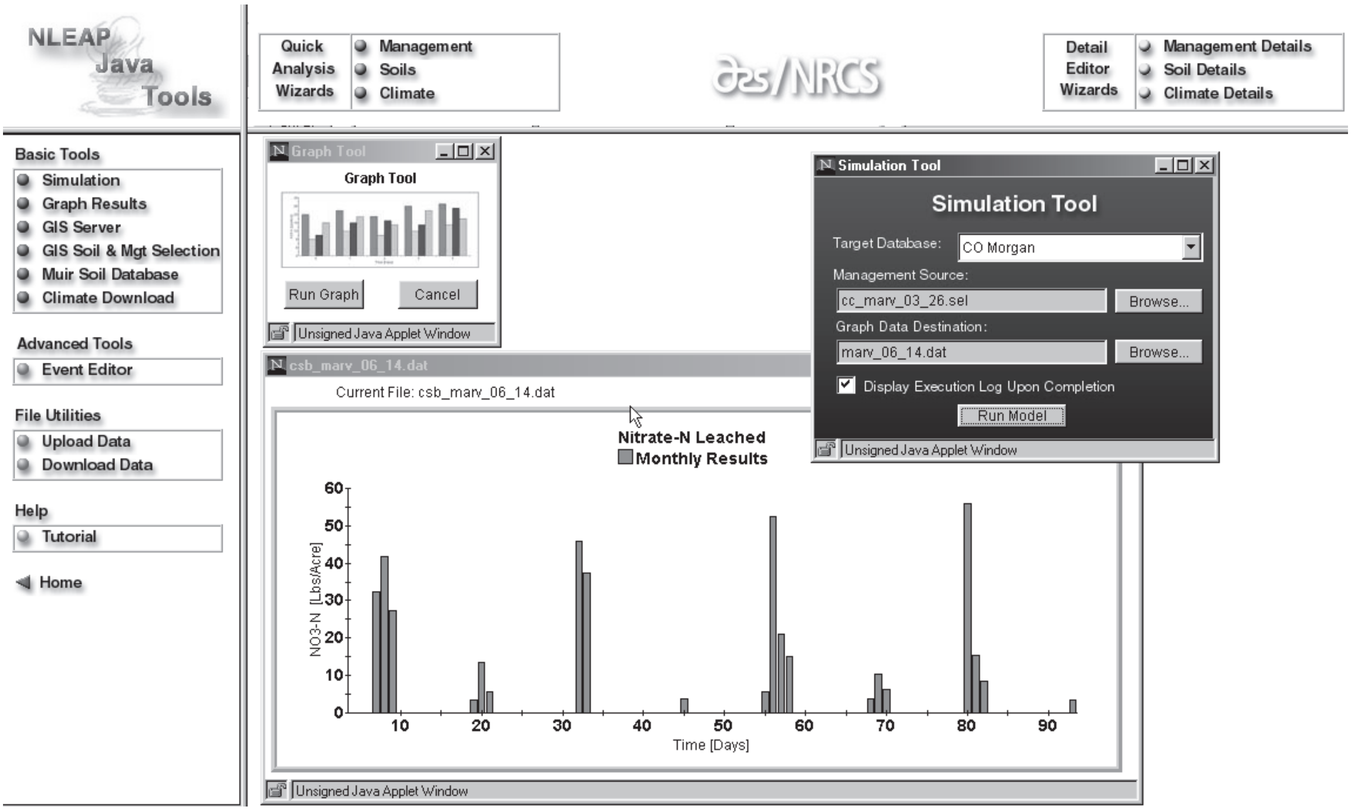

**FIGURE 3.** NLEAP Java applet tools window. The simulation and graphics tools have been loaded and a graph of $NO_3$-N leached has been created.

one provided with Microsoft Access. For example, a user might select a general scenario such as a wheat-corn-fallow (W-C-F) crop rotation with no-till tillage, a starter and side dress fertilizer, and rain-fed conditions. The detailed editor can then be used to adjust individual fertilizer application rates and projected crop yields, or the Microsoft Access editor can be used in conjunction with the detailed editor to develop an entirely new scenario.

## Simulation Server

The simulation server uses existing NLEAP technology developed for the DOS versions of NLEAP and programming from the object-oriented framework of the Great Plains Framework for Agricultural Resource Management (GPFARM) Decision Support System (DSS) for farms and ranches[31]. The simulation server is accessed via the simulation applet tool (Fig. 3). The server consists of Formula Translation (FORTRAN) language soil-process modules adapted and extended from NLEAP DOS versions 1.2 and 1.3[4,6] and linked directly to an object-oriented C++ computer-language framework adapted from GPFARM[32]. Processes simulated include infiltration and transport of soil water and nitrates; carbon and N cycling and transformations on the soil surface and within the soil profile; surface runoff of water, nitrate, and ammonium; nitrate leaching from the root zone; crop uptake of nitrate and ammonium; denitrification losses (including $N_2$ and $N_2O$); and ammonia volatilization. Additional technical details are described in Shaffer et al.[32], Ma and Shaffer[8], and Xu et al.[22]. The simulation server is designed to run multiple years of management and climate scenario data at the option of the user. A linkage is maintained with the GIS server through the field and soil mapping unit (MU) identification codes. This allows the user to selectively run and analyze individual fields and soils or field/soil groups within a study area. Output from the server is returned to the user's account directory for optional plotting (Fig. 3), viewing as data tables, printing, or joining to the GIS attribute file for map displays.

## Graphics Server

Fig. 3 contains a bar graph of monthly $NO_3$-N leached for a corn-sugar beet crop rotation over an 8-year period. This graph was generated using the NLEAP graphic server tool. This server plots user-selected files of $NO_3$-N leached, residual soil $NO_3$-N, crop N uptake, $N_2O$ soil emissions, N denitrified, and N contained in surface runoff. These files are created when the NLEAP simulation server is run and potentially give the user a detailed view of trends during the simulation. They are particularly useful in determining when the system has reached dynamic steady state and in establishing associated values for steady-state conditions.

## GIS Server

The GIS server[32] is based on a customized version of Environmental Systems Research Institute (ESRI) ArcIms 3.0[30] that allows the user to draw field boundaries and then intersect these boundaries with the NRCS Soil Survey Geographic (SSURGO) soil survey map layer for the site. A list of soil mapping units (MUs) within each field is then passed to the NLEAP interface for processing. The user next associates an appropriate management scenario with each soil in the field and calls the NLEAP server. The NLEAP model places results for the last year of the simulation along with other optional parameters for each soil in the field into a Microsoft Access output database table (see graphics server discussion above for list). The GIS interface contains a control button that tells the GIS server to join the new results in the output table to the existing GIS soil attribute table. Model input variables and simulated results are then available for display on the GIS map of the field, as shown in Fig. 4. The ArcIms interface allows display of the soil attributes as individual results or mapped as user-specified ranges across the field. Maps can be printed to the user's local printer or saved as a data layer on the user's computer.

## Database Servers

Using C++ server programs and FTP file transfers, servers were developed to automatically connect with NRCS soil and climate databases, download user selected data subsets, convert these subsets to NLEAP format, and store the results into the proper database tables on the user's account.

## Soils Databases

The NRCS National Soil Information System (NASIS) is the platform that NLEAP will eventually address to access soil-related data. NRCS is in the process of developing the interface for computer simulation models to access NASIS data and download needed information. In the interim, NLEAP is using the older NASIS predecessor, the Map Unit Interpretation Record (MUIR). A Java interface applet allows the user to select the required MUIR data set from the national list. These data are then automatically downloaded to the server and converted to NLEAP format using a C++ program. The same program also clears the data set of partial records and calculates missing values for Cation Exchange Capacity (CEC) and soil water content at 15 bars before writing to the soils database table.

## Climate Database

The NRCS National Water and Climate Center (NWCC) is the clearinghouse for climatic information within the USDA. NWCC, in cooperation with the Unified Climate Access Network (UCAN),

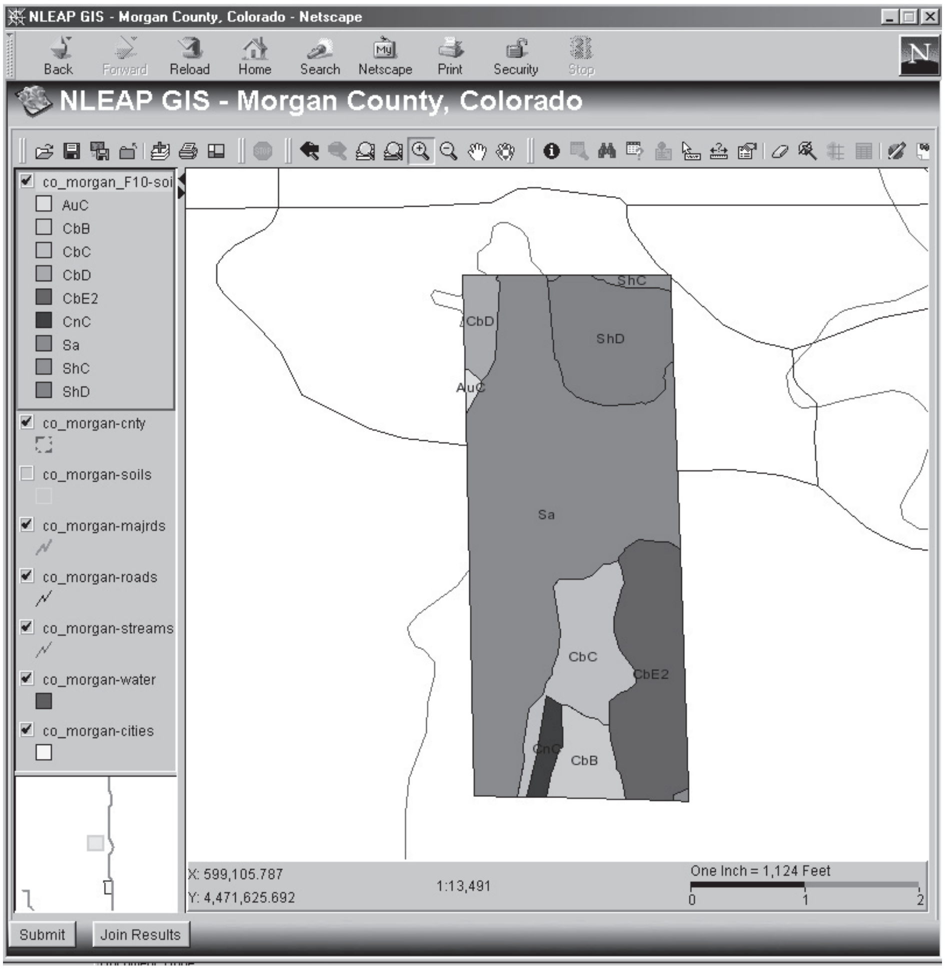

**FIGURE 4.** ArcIms GIS server interface. Soil mapping units are displayed across a field in Morgan County, CO.

a joint project of a number of agencies with the primary architects, the NRCS and Regional Climate Centers, has developed a Web interface to retrieve climate data for a variety of uses. The current version of NLEAP will use the data derived from UCAN. Ultimately, NLEAP will be interfaced with UCAN to provide the required climatic information. In addition, the NWCC is currently working on a project that will provide the basic evapotranspiration information required in most process models to describe water extraction from the soil by plants. These calculated values will improve the estimates provided by process models for runoff, soil loss, and environmental impacts such as nitrate leaching as provided in the NLEAP model.

Currently, NLEAP interacts with the NWCC Climatic Database, allowing the user to access historic and real-time climatic data records from a choice of nearby weather stations through the NWCC FTP site (ftp://ftp.wcc.nrcs.usda.gov/support/climate/daily-data). Temperature and precipitation data are readily available from this Web site; however, potential evaporation data must be obtained by other means. Work is still ongoing to develop options for daily evaporation data for NLEAP. Meanwhile, users must either supply their own potential evaporation data or allow the climate interface to calculate daily potential evaporation from extraterrestrial radiation and air temperature, based on a method developed by Hargreaves and Samani[33].

An interface applet provides the user with a list of available climate stations for each state. The selected climate database file is then downloaded to the server where a C++ program removes data years containing incomplete or missing records, converts the data to NLEAP format, optionally calculates potential evaporation, and writes the resulting climate records to an NLEAP climate database table chosen by the user.

## NRCS Application

NRCS is supporting the development and implementation of this application. It is expected to be used by agency employees and private consultants to prepare nutrient management plans for landowners. Nutrient management plans are increasingly being required by state and federal regulations in response to concern about eutrophication of surface waters and contamination of ground water. Approximately half a million such plans are expected to be prepared in coming decades.

NRCS plans on using NLEAP at the NRCS state office level for watershed planning purposes, and at the field office level for nutrient management planning purposes. With the help of the NRCS GIS planning capabilities associated within the Customer Service Toolkit (TOOLKIT) program and within NLEAP, vulnerable areas in certain hydrologic watersheds can be identified as potentially contributing to nitrate leaching toward ground water, based on cropping systems and nutrients applied. Also, certain areas within individual agricultural fields can be identified as particularly vulnerable to nitrate leaching. Results from the NLEAP output could be used to develop better nutrient management plans, and alter nutrient applications from organic, inorganic, or irrigation sources.

The use of NLEAP to predict airborne losses of N to the atmosphere has great potential for N management. This option will be explored with future funding.

## Management Application Example

The NLEAP Internet client-server system was used to quickly determine how $NO_3$-N leaching from different soils in a field would change in response to a reduction in amount of applied irrigation water. In this test scenario, the user had available soil survey map layers for local NRCS SSURGO soils data, a previously downloaded NRCS MUIR soils attribute file and NRCS NWCC climate database files, and a long-term management scenario for the default cropping system of continuous irrigated corn. This type of information would typically be available in an NRCS state or field office. The field boundaries were drawn on a base map layer of the county roads and then intersected with the NRCS SSURGO soils mapping layer. This produced a list of soil mapping units that was written to a file accessible by the GIS Soil and Management Selection applet (Fig. 5). This applet allows the user to associate predefined management scenarios with each soil in the field. In this instance, we assigned the same continuous irrigated corn scenario to each soil. Precision agriculture applications would involve multiple scenario assignments. After saving this management suite for the field, the simulation applet (Fig. 3) was then used to select and run all the soils in the field for 14 years. The bar graph (Fig. 6a) shows the monthly results for $NO_3$-N leached over this period for the Colby loam soil. Note that annual dynamic steady state with respect to $NO_3$-N leached was reached after about 9 or 10 years at about 42 kg N/ha/year for the irrigation rate of 71 cm/year. Steady state was determined when a running average of annual $NO_3$-N leached for the past 3 years changed less than 10%.

The same set of simulations was then repeated except that the irrigation amounts were reduced by 25% to 53 cm/year. The monthly $NO_3$-N leaching results for this scenario are plotted in Fig. 6b. The annual steady-state values for $NO_3$-N leached of about 15 kg N/ha/year are lower than those shown in Fig. 6a for the higher irrigation rate. Although N input to the system was the same in both cases, the lower irrigation rate allowed a higher crop N uptake efficiency and therefore a reduced value for steady-state leaching of $NO_3$-N. In general, the amount of water applied controls the concentration of $NO_3$-N in the steady-state leachate, but not the mass except in cases such as this where modified leaching of N also impacts another sink[19]. The time to steady state is related to the amount of water applied, with higher irrigation amounts producing shorter times to steady state and vice versa.

## SUMMARY AND CONCLUSIONS

Development of an effective client-server based system for operation of water quality models such as NLEAP on the Internet in conjunction with database, GIS, and graphics servers proved to be feasible given current bandwidth restrictions and provided Java applet sizes did not exceed about 1.5 MB. Java applets were effectively and efficiently used as interface windows in conjunction with a development environment such as Visual Café. Security restrictions associated with Java applets proved to challenging, but were soluble once the security system was understood.

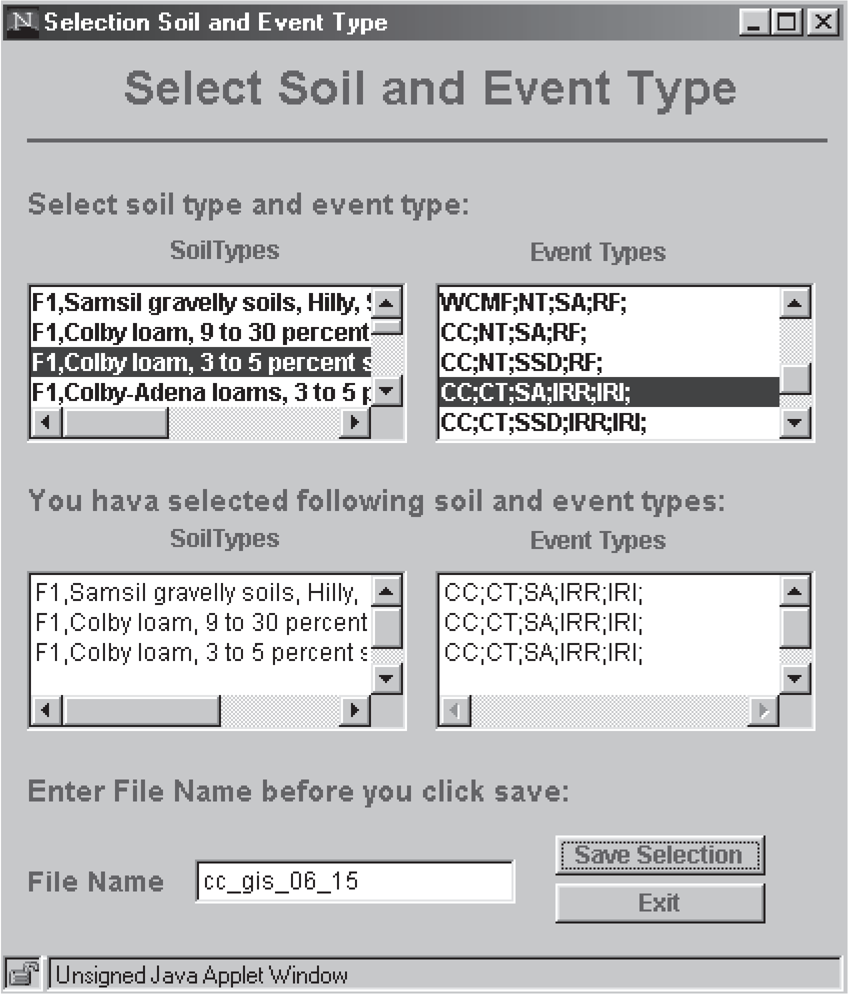

**FIGURE 5.** Association of soils (left side) with available management scenarios (right side).

Download linkages with NRCS soil and climate databases were accomplished using FTP and Telnet protocols and techniques. These techniques proved to be fast and reliable. Similar methods were used to move commands between the user interface and the NLEAP simulation server.

Development of a customized ArcIms interface that allowed drawing of field boundaries and linkage with other GIS tools for intersecting data and joining of data tables proved to be challenging given the development status of the ArcIms system. Integration of the NLEAP simulation model with the ArcIms GIS system was kept relatively easy by using Microsoft Access databases to store key input and output attribute data for display by ArcIms and for access by the NLEAP server. Key links with the ArcIms polygon (shapefile) data were maintained within the attribute files. Transfer of information between the user interface (client) and the servers was kept to a minimum by extensive use of preprogrammed management scenarios in conjunction with the soil and climate databases.

In general, the most difficult part of on-farm simulations is the management event setup. Preset management for a range of common crop rotations together with rapid on-line editing of selected key parameters shows promise for solving at least part of this major problem. An improved client applet is being developed that will allow the user to drag and drop management icons over a timeline to help set up the management event database. A rule-based system that will allow the user to specify general rules for managing each farm field rather then having to specify each management event is also under development.

Other major challenges encountered during the development of this Internet application were the rapidly changing programming environment and security concerns. The application maintains separate accounts for individual users and passes information back and forth across numerous machines. Minimizing computer security vulnerabilities continues to be a major challenge.

## ACCESS TO THE INTERNET NLEAP WEB SITE

General access to the Internet NLEAP site is available at http://nleap.usda.gov/nresearch.html. The home page provides general information about the application, an on-line tutorial, and a list of NLEAP references. Access is also provided to the NLEAP Java applet tools needed to run the application, but the potential user will first need to establish an account on the server. This can

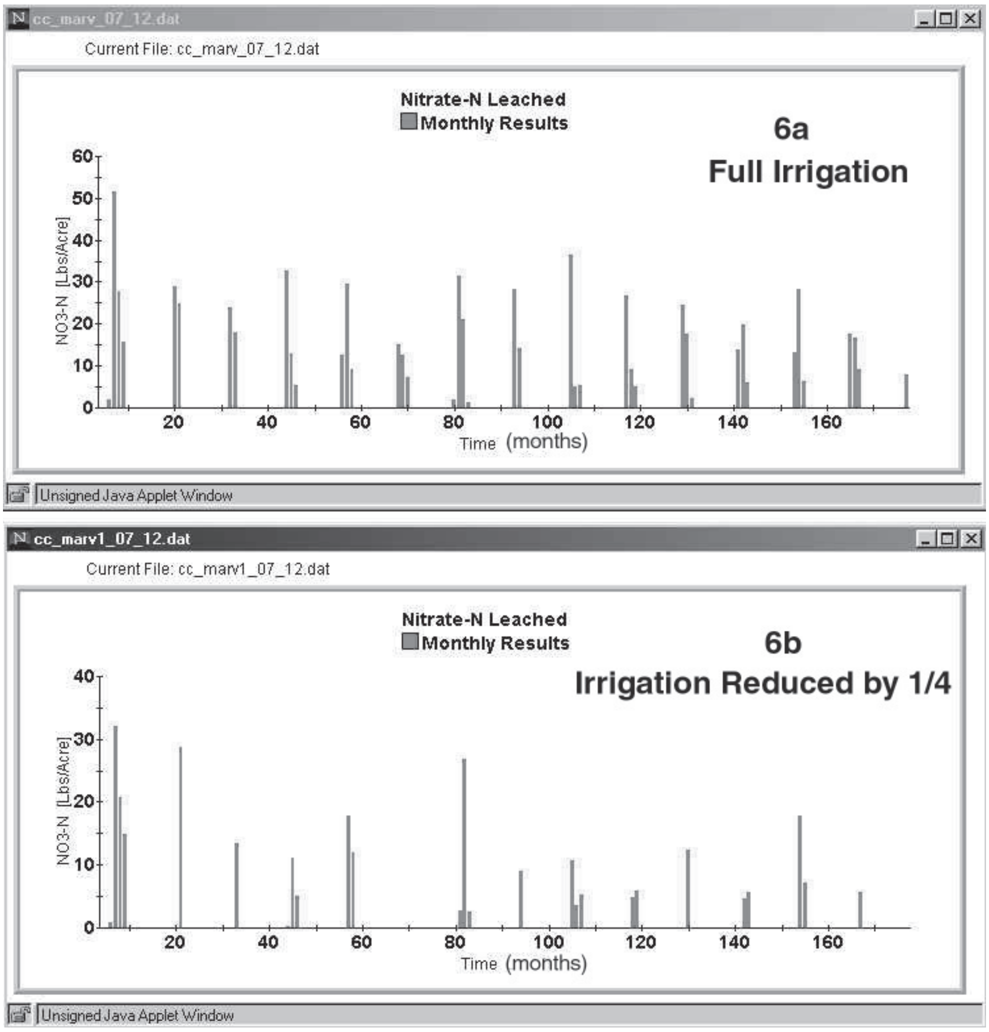

**FIGURE 6.** Monthly simulated $NO_3$-N leached for Colby loam under (a) full irrigation and (b) irrigation reduced by 25%. Bars represent months where leaching occurred below the crop root zone.

be initiated by contacting the authors at the e-mail address shaffer@gpsr.colostate.edu.

| | | |
|---|---|---|
| Received: | September | 18, 2001 |
| Revised: | October | 15, 2001 |
| Accepted: | October | 29, 2001 |
| Published: | November | 14, 2001 |
